# Touching-bone acupuncture in the treatment of chronic pain: A protocol for an overview of systematic reviews and meta-analysis

**DOI:** 10.1097/MD.0000000000027195

**Published:** 2021-11-19

**Authors:** Xiaoping Li, Wan Wei, Yuan Wang, Qiang Wang, Zhibin Liu

**Affiliations:** a The Third Clinical Medical College, Zhejiang Chinese Medical University, Hangzhou, Zhejiang, China; b School of Acupuncture-Moxibustion and Tuina, Shanghai University of Traditional Chinese Medicine, Shanghai, China; c Innovation Research Center of Acupuncture and Medicine, Shaanxi University of Chinese Medicine, Qindu District, Xianyang, Shaanxi, China; d Shaanxi Key Laboratory of Acupuncture and Medicine, Qindu District, Xianyang, Shaanxi, China.

**Keywords:** chronic pain, meta-analysis, touching periosteum acupuncture therapy

## Abstract

**Background:**

Chronic pain is the most common disease in the world, which lead the patients to suffer the disability both physically and psychologically. The chronic pain can affects the patients to work, socialize, sleep and can lead to depressive illness, decreased motivation, and a reduction in physical activity. Acupuncture is a promising treatment for the chronic pain which has a long history of use in China. This protocol aims to assess the effectiveness and safety of touching periosteum acupuncture therapy on patients with chronic pain.

**Methods:**

Randomized controlled trial literatures which include touching periosteum acupuncture therapy for treating chronic pain will be searched from 8 electronic databases including China Biology Medicine disc, VIP database, WanFang database, China National Knowledge Infrastructure, PubMed, Cochrane Library, Excerpt Medical Database, and Web of Science. The language will be restricted to Chinese and English. The primary outcome is to measure the relief of the pain by Visual Analogue Scale. Two or 3 reviewers will conduct the study selection, data extraction and the evaluation of the risk of bias. RevMan software (V.5.3) will be used to perform the assessment of the risk of bias and data synthesis.

**Results:**

From this study, we will confirm the effectiveness of safety of in the treatment of chronic pain.

**Conclusions:**

We will ascertain the effectiveness of safety of touching periosteum acupuncture therapy in the treatment of chronic pain, to provide evidence to guide touching periosteum acupuncture therapy for patients with chronic pain in the future.

**Ethics and dissemination:**

Ethics approval will not be necessary, because the included publications in our study are all from published articles. This systematic review will be published in a peer-reviewed journal or conference report to provide a reference in this field.

**Trial registration:**

CRD42021243387.

## 1 Introduction

Chronic pain is the most common and consequential disease in the world.^[1]^ The World Health Organization recognizes chronic pain as the secondary disease of long-term conditions.^[2]^ Chronic pain is a leading source of patients suffering the disability both physically and psychologically.^[3,4]^ The International Association for the Study of Pain have defined chronic pain as that which lasts for longer than 3 months.^[5]^ Chronic pain is not an immediately life threatening condition; people with chronic pain continue to live. Individuals living with pain often experience a very poor quality of life, it affects their ability to work, socialize, sleep and can lead to depressive illness, decreased motivation and a reduction in physical activity.^[6]^ Pain is one of the most important reasons for patients to seek medical care, as such, chronic pain represents a major challenge for health service provision and government policy.^[7]^

There are pharmaceutical and nonpharmaceutical treatments for chronic pain. Treatment choices include medications, surgical, psychological therapy, physical therapy, and alternative complementary therapy which include acupuncture and moxibustion. As one of the most important methods in traditional Chinese medicine, acupuncture has been used to treat chronic pain in China for centuries, and some researchers have demonstrated that acupuncture has promising efficacy in the treatment of chronic pain.^[8–11]^ Acupuncture typically involves the insertion of thin solid needles into specific points in the body (acupoints).^[12]^ Once needles are inserted, they may be stimulated manually or electrically.^[13]^ A key issue of acupuncture research is the notion of “dose”.^[14]^ It is unknown what the optimal dose of acupuncture is and even more importantly, how to classify dose. Does dose include the depth of penetration, the frequency of treatments, the number of needles, the amount or type of stimulation? Recent studies suggests that the depth of penetration matters and the touching periosteum acupuncture therapy technique may be superior to the acupuncture of usual depth.^[15,16]^

The touching periosteum acupuncture therapy technique refers to deep needling method, originated from the short needling and Shu needling of the ancient needling methods recorded in the LingShu GuanZhen. The target points are the reaction sites on meridian near to bone. During the needle insertion, the needle tip is thrust deeply into the bone or the needle body is closely attached to the bone so as to stimulate periosteum specifically. This needling technique contributes to the satisfactory effect on spasmodic, deep-located and intractable pain disorder, motor system diseases, mental diseases and cerebral diseases, etc. The mechanism can be that the polymodal receptors distributed in the periosteum stimulated by acupuncture can induce analgesic effects through activation of opiate or nonopiate mediated endogenous pain inhibitory systems.^[17–19]^

Even though widely used in today's clinical practice,^[20]^ the optimal dose of acupuncture has remained a controversial issue. As the effectiveness and safety of touching periosteum acupuncture therapy for treating chronic pain remain uncertain and current evidence is limited. Moreover, to date, there are no systematic reviews to evaluate the efficacy of touching periosteum acupuncture therapy in the treatment of chronic pain, so a critical examination of the evidence regarding touching periosteum acupuncture therapy for treating chronic pain is warranted.

In our study, we will perform the first systematic review and meta-analysis to investigate the efficacy and safety of touching periosteum acupuncture therapy on the treatment of chronic pain to provide evidence to confirm the current controversy about the touching periosteum acupuncture therapy on the treatment of chronic pain.

## 2 Methods

### 2.1 Design and registration of the study

This protocol will be conducted according to the guideline of the Cochrane handbook for systematic reviews of interventions. This protocol has already been recorded in the prospective international registry of systematic review^[21]^ and the trial registration number is CRD42021243387. (https://www.crd.york.ac.uk/PROSPERO/display_record.php?RecordID=243387)

### 2.2 Inclusion criteria for study selection

#### 2.2.1 Type of studies

Only randomized controlled trials (RCTs) will be included in this review. Language of literature will be limited to Chinese and English. Additionally, other designs such as in vivo, in vitro, case report, and non-RCTs will also be excluded.

#### 2.2.2 Type of participants

Participants diagnosed with chronic pain will be Included, regardless of gender, age, race, or nationality, who received acupuncture therapy (with or without other treatment) will be included. Besides, patients with other serious illnesses, such as cancer, cardiovascular disease, and liver and kidney disease, will be excluded.

#### 2.2.3 Type of interventions

We will include studies evaluating the following treatments including manual acupuncture or electro-acupuncture or warm/fire acupuncture with the insertion of needles at the acupuncture points and the tips of the needles must touch the periosteum. And the control group received the superficial needling at the acupuncture points but without touching the periosteum.

### 2.3 Outcome measures

#### 2.3.1 Primary outcomes

The primary outcome measure will be the Visual Analogue Scale.

#### 2.3.2 Secondary outcomes

1. The proportion of patients achieving at least a 50% reduction in the intensity and frequency of pain relief,
2. Quality of life,
3. Adverse events,
4. The time needed to obtain pain relief: complete pain relief or any small amount of pain relief.

### 2.4 Study exclusion criteria

Participants were diagnosed with acute pain will be excluded. Studies using incorrect randomization methods will be excluded. Additionally, other designs such as in vivo, in vitro, case report, and non-RCTs will also be excluded. Patients with other serious illnesses, such as bone tuberculosis, cancer, cardiovascular disease, and liver and kidney disease, will be excluded. Articles comparing different acupoints or different forms of acupuncture will be excluded.

### 2.5 Search strategies

We will retrieve 8 electronic databases from their inception to June 31, 2021, which include 4 Chinese databases: China Biology Medicine disc, VIP database, Wangfang database, China National Knowledge Infrastructure, and 4 English databases: PubMed, Cochrane Library, Excerpt Medical Database, Web of Science. The language will be restricted to Chinese and English. The search strategy for PubMed is shown in Table 1. We would use the equivalent search words in Chinese databases.

**Table 1:** Search strategy used in PubMed database.

No.	Search items
1	Acupuncture
2	Acupuncture therapy
3	Electroacupuncture
4	Warm acupuncture
5	Fire acupuncture
6	Needling
7	needles
8	1 or 2–7
9	Pain
10	Chronic pain
11	Analgesia
12	9 or 10–11
13	Touching bone
14	Bone nearby
15	Touching periosteum
16	13 or 14–15
17	Randomized
18	Randomized controlled trial
19	Randomly
20	Clinical trial
21	17 or 18–20
22	8 and 12 and 16 and 21

### 2.6 Study selection

**Figure 1:**
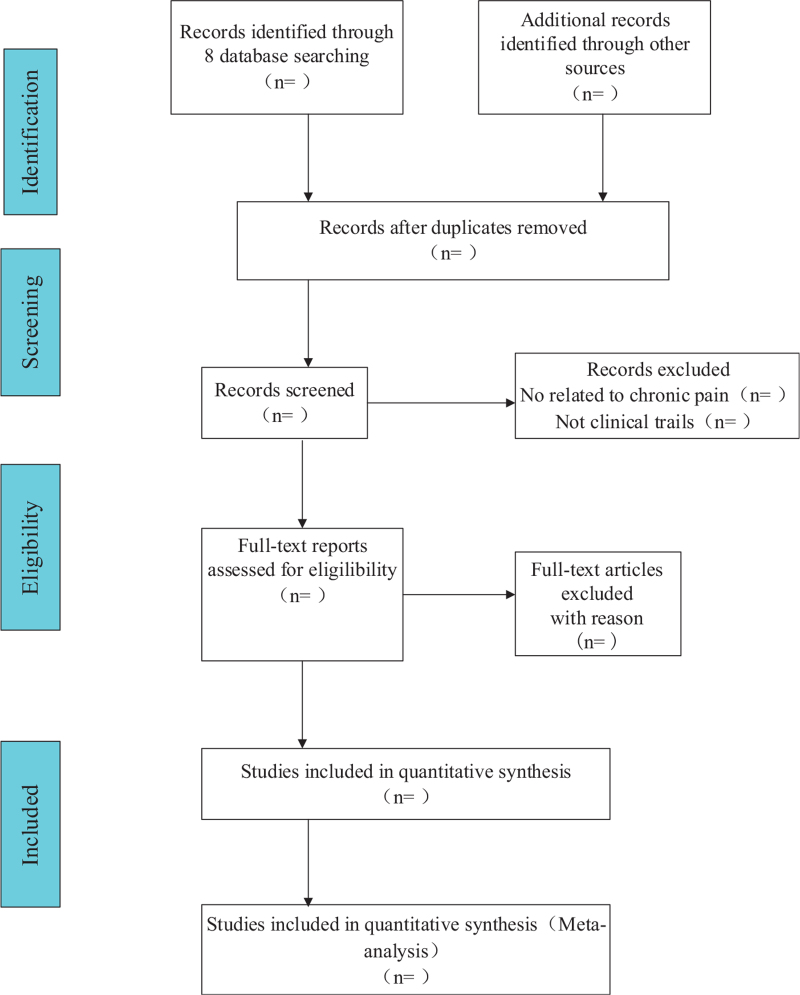
Flow diagram of the study selection process.

According to the inclusion criteria, all retrieved studies will be assessed by 2 reviewers (XPL and WW). The title and abstract of all studies will be evaluated, respectively. Any duplicate studies will be removed. After title and abstract screening, the full-text copies of all eligible studies will be downloaded for re-evaluation. Once the reviewers are uncertain about the eligibility of any study, a third reviewer (LZB) will be consulted. Excluded studies and the reasons of exclusion will be recorded. The specific process of study screening will be displayed in a preferred reporting items for systematic reviews and meta-analyses. The flow diagram of all study selection procedure is shown in Fig. 1.

### 2.7 Data extraction

Two reviewers (XPL and YW) will independently extract the following information from each included study: first author, publication year, country, sample, study design, interventions, comparisons, outcome measures (primary and secondary outcomes), risk of bias assessment, and results. Any disagreement will also be solved by introducing a third researcher for judgment.

### 2.8 Missing data management

We will contact the original author to obtain the missing or incomplete data and will wait 1 month after an email is sent to receive a reply. If we are unable to obtain the missing data, the incomplete data will be excluded from the analysis.

### 2.9 Assessment of risk of bias

Two authors (YW and QW)of this review will assess the risk of bias of the included studies, by using the Cochrane Collaboration tool.^[22]^ The following domains for risk of bias will be evaluated: sequence generation, allocation sequence concealment, blinding of participants and personnel and outcome assessors, incomplete outcome data, selective outcome reporting, and other sources of bias. The judgment on these items will be classified into 3 levels: “low risk of bias”, “high risk of bias”, or “unclear risk of bias”.^[23]^ The conflicts or any discrepancies will be resolved by discussion or will be judged by another reviewer to achieve the consensus.

### 2.10 Measures of treatment effects

Clinical data will be imported into the RevMan software (V.5.3, Cochrane) to perform data synthesis. The dichotomous data will be analyzed using a risk ratio with 95% confidence interval. The mean difference or standard mean difference with 95% confidence interval will be used to analyse the continuous data. If different evaluation tools are used, standard mean difference will be used.

### 2.11 Assessment of heterogeneity

The heterogeneity will be assessed by I^2^ statistical test. If the I^2^ test <50%, the fixed-effect model will be used for data synthesis.^[24]^ And the random-effects model will be conducted with heterogeneous data, where I^2^ test is between 50 and 75%. If the I^2^ test is higher than 75%, we will find the possible reasons from both clinical and methodological perspectives and provide an explanation or conduct subgroup analysis.

### 2.12 Assessment of reporting bias

A funnel plot will be generated to assess reporting bias when more than 10 trials are included.^[25]^ Otherwise, STATA V.15.1 software will be used to perform the Egger test.

### 2.13 Data synthesis

Clinical data will be imported into the RevMan software^[26]^ (V.5.3) to perform data synthesis, and the significance threshold will be *P* < .05 on 2 sides. A forest plot for each parameter will be constructed to indicate the weight ratio of each incorporated study.

### 2.14 Sensitivity analysis

Sensitivity analysis will be conducted to monitor the robustness of primary decision made in the review process. The lower quality trials will be excluded to repeat the meta-analyses to assess quality when significant statistical heterogeneity arises. The results of the sensitivity analysis will be presented in summary tables. The risk of bias in the review process as indicated by the results of the sensitivity analysis will be discussed.

### 2.15 Subgroup analysis

Subgroup analysis will be conducted if data is available. Variations will be considered in the characteristics of the treatments, participants, control types. And there will be subgroups to interpret the heterogeneity.

### 2.16 Quality of evidence assessment

According to grading of recommendations assessment, development, and evaluation method,^[27]^ the quality of the evidence can be regarded as 4 levels: high quality, moderate quality, low quality, and very low quality.^[28]^

### 2.17 Ethics and dissemination

The aim of this meta-analysis of RCTs is to evaluate the efficacy and safety of touching-bone acupuncture in the treatment of chronic pain. Ethics approval will not be necessary, because the included publications in our study are all from published articles. This systematic review will be published in a peer-reviewed journal or conference report to provide a reference in this field.

## 3 Discussion

Chronic pain has a high prevalence, and greatly affects the quality of life of the patients. Acupuncture is a promising treatment for the chronic pain which has been widely used in clinical. But the touching periosteum acupuncture therapy for treating chronic pain remain uncertain and current evidence is limited. This protocol aims to assess the effectiveness and safety of touching periosteum acupuncture therapy on patients with chronic pain to provide relatively convincing evidence for this acupuncture technique for people with chronic pain, physicians and policymakers.

## Acknowledgments

The authors would like to express their gratitude to all the advisors of this study.

## Author contributions

**Conceptualization:** Xiaoping Li, Wan Wei.

**Data curation:** Xiaoping Li, Qiang Wang.

**Formal analysis:** Xiaoping Li, Wan Wei, Qiang Wang.

**Funding acquisition:** Zhibin Liu.

**Investigation:** Yuan Wang.

**Methodology:** Xiaoping Li, Wan Wei.

**Resources:** Qiang Wang.

**Software:** Xiaoping Li, Wan Wei.

**Supervision:** Yuan Wang, Zhibin Liu.

**Validation:** Wan Wei.

**Writing – original draft:** Xiaoping Li, Wan Wei.

**Writing – review & editing:** Xiaoping Li, Wan Wei.
